# Core food group intake in people with enduring psychotic illness accessing community mental health services

**DOI:** 10.1371/journal.pmen.0000540

**Published:** 2026-07-09

**Authors:** Premala Sureshkumar, Rebecca Lancaster, Tim Lambert

**Affiliations:** 1 Department of Psychiatry, Concord Medical School, University of Sydney, Sydney, Australia; 2 Living Well, Living Longer Program, Sydney Local Health District, Sydney, Australia; 3 University of Sydney and Sydney Local Health District, Sydney, Australia; National University of Singapore, SINGAPORE

## Abstract

Poor dietary intake in people with enduring psychotic illness (EPI) is related to physical comorbidity which in turn raises the risk of premature death due to cardiovascular disease. This study evaluates the dietary intake of the five core food groups in consumers with EPI attending a dedicated multidisciplinary clinical service, the Collaborative Centre for Cardiometabolic Health in Psychosis (ccCHiP). As part of routine care, dietary intakes were collected from a sample of consecutive adult consumers attending the ccCHiP service during May 2014 through December 2020. In total, 1484 occasions of service were provided to 1084 consumers (mean age 44.3, SD 12.7 years and 63% males). Thirty-one percent of those assessed, met the recommended daily fruit intake according to the Australian Dietary Guidelines (ADG). In nine percent of assessments, people met the recommended daily vegetable intake. The proportions of people meeting daily intake of protein, dairy, and grains were 48, 20 and 49 percent respectively. On multivariable analysis, males, those with a sedentary lifestyle, receiving pension benefits, and living outside the family were statistically significantly associated with lower vegetable intake. Similarly, males, those with a sedentary lifestyle, smoking, and those living outside the family, particularly in a boarding house, were statistically associated with lower fruit intake. There was a significant gap for fruit intake compared to population norms, with this gap widening with age. Moreover, a significant proportion of consumers reported no vegetable (7%), or fruit (34%) intake. Both were significantly lower than Australian norms. On average, consumers with EPI under-consumed core food groups, in particular, they consumed less fruit daily compared to the Australian General population. These findings suggest that to improve the parlous rates of premature cardiovascular mortality in those with EPI, increasing dietetic input into formulating preventative health interventions should be strongly considered when planning interventional services.

## Introduction

People living with enduring psychotic illness (EPI) [1] experience high rates of physical health comorbidity, with up to a 30-year reduction in life expectancy compared with the general population [2–7]. In those experiencing EPI, premature mortality rates are highest in people with schizophrenia – 2 to 2.5 times greater than the general population [8,9]. This *“scandal of premature mortality”* [10] is largely attributed to cardiometabolic disease, with evidence suggesting that this longevity gap is increasing [8,11–13]. Poor diet is recognized as one of the key modifiable risk factors driving these physical health disparities, alongside other lifestyle behaviours such as tobacco-use and physical inactivity [12,14].

The interplay between mental health, physical health, and diet is complex. Dietary intake and eating behaviour are influenced by the orexigenic effects of many psychotropic medications resulting in increased appetite, reduced satiety, and enhanced cravings for high energy foods [15]. Poor food choices are also influenced by food insecurity [16], low health literacy [17], cognitive impairment, and the negative symptomology that is characteristic of EPI, such as schizophrenia. In turn, research has also demonstrated the link between nutrition and mental status as being bidirectional, with diet now recognized to play a role in both prevention and treatment of mental illness [18,19]. Addressing dietary risks therefore has the potential to improve both mental and physical health outcomes in this vulnerable population.

Within the general population, it is widely accepted that poor diet quality is associated with adverse physical health outcomes, including cardiovascular disease and premature mortality [20,21]. The 2013 Australian Dietary Guidelines (ADG) and the Australian Guide to Healthy Eating (AGHE) [22] were developed using the latest evidence and expert opinion to help in the prevention of diet-related chronic disease to improve health and wellbeing of the Australian community. Evidence suggests that each additional serve of fruit or vegetables is associated with a reduced risk of coronary heart disease and stroke, with protective effects stronger at higher intake (up to 5 serves daily) [22]. Very few Australian adults are meeting guidelines for the recommended daily intake of fruit and vegetables, with high proportion of total daily energy intake coming from discretionary food and drinks [23]. A systematic review and meta-analysis of dietary intake in psychotic disorders found that adults with EPI generally consume a diet higher in energy and sodium, which is likely associated with increased hunger and preference for convenience foods and sugary drinks [24]. Results from this review also highlighted that to date, no dietary-assessment method or tool has been thoroughly validated in the EPI context., Only a small number of these studies have specifically explored food group intakes amongst adults with EPI, with nine studies reporting low fruit and vegetable intake compared to country-specific recommendations [24]. These were relatively small-scale studies, with the exception of Hahn et al (n = 1286), which utilized information from the Australian National Survey of Psychosis (2010) [25]. This included 5 questions about diet and required the user to have an adequate understanding of standard serves so as to be able to accurately estimate intake. A more recent questionnaire survey in England estimated that up to 85% of people with schizophrenia and bipolar disorders consumed less than the five recommended portions of fruits and vegetables per day [26].

The increasing recognition and inclusion of dietitians as part of routine mental health care provides the platform to further our understanding of dietary intake patterns amongst this cohort.

An example of this being the Collaborative Centre for Cardiometabolic Health in Psychosis (ccCHiP), which provides a one-stop-shop, multidisciplinary, consultative service for patients with EPI who are referred from local community mental health centres and general practitioners [27].

In this study, we aim to explore the consumption patterns of the core food groups in people with EPI, which is essential to better describe the nutritional disparities that contribute to their poor physical health. Furthermore, we investigate the factors associated with their poor dietary intakes. While it has been identified that people with EPI generally consume less food from core food groups, differences from the general Australian population remains to be further explored.

It is hoped that information from dietary assessments collected as part of the clinic will provide possible targets for people with EPI to improve diet quality and therefore their physical health. Exploring food groups, rather than specific nutrient targets, can be translated more readily into practical, strength-based goals to improve physical health.

## Methods

### Ethics statement

Collection and analysis of ccCHiP data were approved through the Sydney Local Health Area District ethics committee (REGIS 2021/ETH00456 (Previously HREC/16/CRGH/101). A waiver for informed consent was obtained as the study was performed on de-identified routinely collected clinical data and carried minimal risk to participants.

This retrospective observational study included adults (aged 18–65 years) with EPI who attended the ccCHiP clinic between May 2014 and December 2020. Data used for this research study were accessed during 13^th^ October 2022 and 9^th^ August 2023.

Enduring psychotic illness was defined to refer to ‘people with recurrent psychoses associated with schizophrenia and other psychotic illnesses’ [1]. When the patients are referred to the ccCHiP clinic, the diagnosis is already made by the referring doctor. The ICD_10 coding system is used in Sydney Local Health District for diagnostic categories.

Within a single session, the ccCHiP clinical service integrates clinical assessments from a psychiatrist, endocrinologist, cardiologist, exercise physiologist, sleep clinician, oral health team, and dietitian [27]. As part of the multidisciplinary assessment, a dietitian specialising in the care of mental health patients provided dietary assessment and counselling. The dietitians followed a standardized procedure and participated in regular feedback meetings, ensuring consistent collection of diet history data. As part of standard care, a diet history is undertaken in the first instance, utilising food models and pictures to assist with quantifying intake along with a checklist for commonly forgotten items (including specific drinks [28] and fast foods). If diet history was unable to be conducted due to difficulties with recall, the dietitian would move to a 24-hr recall or food frequency-style assessment. All data were recorded in the clinical service’s bespoke clinical informatics system. The dietitian then estimated the usual consumption of the five core food groups (vegetables, fruits, grains, dairy, and protein), based on serves as defined by AGHE. To remain consistent with the ADG, only non-discretionary food sources were counted towards the amounts of the five food groups. Estimated serves discretionary foods were not included in this study.

In the absence of a validated dietary assessment tool for those living with EPI, the dietitian conducting the assessment estimated the reliability of the responses received from consumers. Based on their clinical judgement, this was recorded as reliable/unreliable. The intention of collecting this information was to serve as an internal communication tool only and to better assist clinicians to manage and assess their physical health needs whilst in the clinical setting.

We conducted an additional subgroup analysis for those that were recorded as ‘reliable’ and results from this sensitivity analysis showed almost the same associations as the main analysis. This loss of significance is likely due to reduced sample size and lower statistical power within subgroup. Importantly, the estimated effect sizes remained similar in direction and magnitude to the main analysis, suggesting that the underlying association is consistent, although less precisely estimated in smaller subgroups.

To identify the factors that may influence the consumption of food groups, we explored the association between demographic factors such as age, gender, body mass index, waist to height ratio, smoking, and socio-economic status.

Data were analysed using SAS version 9.4 (SAS institute, Inc., Cary, NC, USA) [29]. Only patients who were seen by a dietitian were included in the analysis. Descriptive analysis included frequencies and percentages for categorical variables and mean and standard deviations for continuous variables. We included reports of zero (nil) consumption as these provide valid estimates in this cohort. Univariable and multivariable regression analyses were carried out to explore the association between independent variables and consumption of the number of serves of fruits and vegetables. For the purpose of this paper, focus was placed on fruits and vegetables intake only, in order to be able to compare to both Australian population norms and dietary guidelines. In this approach, potential factors associated with reduced consumption of food groups were analysed in a backward selection procedure. A backward selection model was chosen to evaluate all demographic and clinical variables with potential relevance to the outcome, ensuring the key predictors were not missed, resulting in a robust final model. Variance inflation factors were examined to assess potential multicollinearity between predictors. Variables with P values < 0.10 in univariable associations were entered into the multivariable model with a global (type III) p < 0.05 being the criterion for removal of variables. The dependent variable – number of serves – was log transformed to provide a normal distribution because of a right skewed distribution and analysed as a continuous variable. ‘Proc Mixed’ procedure in SAS was used to conduct linear mixed model analysis for the repeated measures data (visits as the unit of analysis) specifying subject as random effect. An autoregressive correlation structure was specified to account for within-subject correlations over time. Model fit was compared using Bayesian Information Criterion values obtained from Proc Mixed procedure in SAS. In this procedure, missing data were handled through restricted maximum likelihood (REML) estimation by default, which allows all available repeated-measures observations to contribute to the model under the missing at random assumption. With the dependent variable, consumption of the number of serves of fruits and vegetables being log-transformed, we express coefficients from the log-transformed model as percentage changes in the outcome using the standard conversion $\left(e^{\beta }-1\right)\times 100$.

The difference between number of serves consumed daily and ADG recommended daily serves (which are age and gender-specific) were calculated. Recommended average standard serves per day for each food group derived by the ADG guidelines were used to categorise the number of people consuming ‘adequate’ and ‘inadequate’ amounts. To discern the difference between adequate intake and higher intake patterns, higher intakes of core food group were defined as ≥1 serve above recommendations.

Dietary intake was compared with the general population from the Australian Health Survey data, 2017–2018 [30]. This was considered the most reflective of general population norms, considering the period over which observational data in this study were collected. The most recent data from the National Health Survey 2020–21 were collected online during the COVID-19 pandemic and is a break in time series and cannot be used in comparison with this study, which was largely completed prior to the impacts of the COVID-19 pandemic in Australia.

## Results

The sample included a total of 2498 occasions where people were seen in the ccCHiP multidisciplinary clinic. Of these, 79% included an interaction with a dietitian with data collected for three quarters of these occasions (n = 1484). This included a total of 1084 consumers (mean age 44.3, SD 12.7 years and 63% males) who attended for their initial assessment and their subsequent visits on 400 occasions. Of those who saw a dietitian, 39% received recommendations to improve their intake of core food groups. The most common psychiatric diagnosis of the ccCHiP attendees was schizophrenia (65%) and 52.0% were current smokers. More than half (64.0%) were on a disability pension and further 20% were unemployed or engaged in studies.

Mean (SD) serves were 2.5 (1.8) for vegetables, 1.2 (1.6) for fruit, 1.7 (1.6) for dairy, 2.5 (1.5) for protein, and 5.2 (2.6) for grains.

Table 1 (n = 1084 consumers) presents the demographics and some factors that may influence the daily consumption of food group intake. On average, females consumed slightly more vegetables (mean 2.7 serves, 54% of target) compared to males (mean 2.4 serves, 40% of target). Similarly, the daily consumption of fruits was higher in females (1.4 serves, 70% of target) vs males (1.2 serves, 60% of target).

**Table 1:** Daily servings (mean + /- SD) of food groups (n = 1084).

	Vegetables	Fruits	Dairy	Grains	Protein
**All occasions of service (n = 1484)** **All consumers (n = 1084)**	2.50 (1.77) 2.51 (1.81)	1.23 (1.64) 1.24 (1.68)	1.70 (1.62) 1.67 (1.53)	5.24 (2.63) 5.27 (2.7)	2.53 (1.53) 2.58 (1.59)
**Age group (years)**					
**18-24**	2.61 (1.79)	1.31 (1.51)	1.19 (1.0)	5.57 (2.70)	3.03 (1.83)
**25-34**	2.65 (2.05)	1.64 (2.68)	1.51 (1.24)	5.32 (2.58)	3.06 (1.63)
**35-44**	2.44 (1.71)	1.28 (1.66)	1.74 (1.61)	5.45 (2.93)	2.49 (1.40)
**45-54**	2.51 (1.94)	1.10 (1.33)	1.74 (1.60)	5.28 (2.70)	2.46 (1.51)
**55-64**	2.43 (1.56)	1.10 (1.14)	1.87 (1.76)	4.89 (2.60)	2.33 (1.73)
**>64**	2.59 (1.65)	1.14 (1.20)	1.34 (0.88)	5.26 (2.42)	2.29 (1.22)
**Gender**					
**Male**	2.38 (1.84)	1.18 (1.74)	1.75 (1.72)	5.66 (2.92)	2.83 (1.69)
**Female**	2.74 (1.73)	1.36 (1.55)	1.53 (1.12)	4.62 (2.15)	2.16 (1.31)
**BMI**					
**Normal**	2.76 (1.72)	1.15 (1.34)	1.38 (1.21)	4.77 (2.40)	2.31 (1.45)
**Overweight**	2.41 (1.64)	1.26 (1.36)	1.62 (1.65)	5.09 (2.70)	2.53 (1.71)
**Obese**	2.48 (1.92)	1.28 (1.95)	1.79 (1.54)	5.55 (2.79)	2.71 (1.56)
**Waist-to-height ratio** ^**Ф**^					
**Normal**	2.67 (1.75)	1.43 (1.59)	1.42 (1.22)	4.72 (2.50)	2.29 (1.56)
**Abnormal**	2.50 (1.82)	1.22 (1.65)	1.69 (1.56)	5.36 (2.74)	2.63 (1.60)
**Psychiatric diagnosis**					
**Schizophrenia**	2.47 (1.83)	1.23 (1.42)	1.74 (1.63)	5.35 (2.75)	2.52 (1.59)
**Schizoaffective**	2.82 (1.88)	1.32 (1.52)	1.62 (1.23)	5.10 (2.62)	2.67 (1.51)
**Bipolar**	2.54 (1.65)	1.17 (1.57)	1.56 (1.42)	5.15 (2.38)	2.54 (1.49)
**Depression**	2.96 (1.82)	1.39 (1.22)	1.59 (1.21)	5.17 (2.73)	2.61 (1.73)
**Other psychosis**	2.41 (1.77)	1.46 (3.06)	1.44 (1.23)	5.51 (2.93)	3.07 (1.78)
**All others**	2.06 (1.58)	0.95 (2.86)	1.40 (1.37)	3.64 (2.06)	2.25 (1.31)
**Vocational level**					
**Employed**	2.65 (1.48)	1.31 (1.27)	1.56 (1.19)	5.15 (2.39)	2.86 (1.46)
**Unemployed**	2.59 (1.80)	1.37 (1.63)	1.66 (1.61)	5.40 (2.74)	2.66 (2.09)
**Pension**	2.39 (1.82)	1.14 (1.73)	1.70 (1.62)	5.28 (2.83)	2.46 (1.45)
**Studying**	2.50 (1.69)	1.69 (2.11)	1.59 (1.22)	4.80 (1.98)	2.73 (1.49)
**Living arrangements** ^**Ф**^					
**Alone**	2.34 (1.67)	1.28 (2.04)	1.84 (1.90)	4.79 (2.62)	2.48 (1.76)
**Spouse/de facto**	3.02 (2.40)	1.53 (1.59)	1.53 (1.0)	5.76 (2.62)	2.81 (1.96)
**Parents or siblings**	2.67 (1.82)	1.36 (1.58)	1.56 (1.27)	5.52 (2.91)	2.87 (1.53)
**Other family member**	2.22 (1.92)	0.85 (1.14)	1.51 (1.37)	5.27 (2.71)	2.43 (1.40)
**Group of others**	2.75 (2.36)	0.90 (0.88)	1.89 (2.21)	4.98 (1.69)	2.36 (1.25)
**One other (not family)**	2.04 (1.80)	1.59 (1.76)	1.63 (1.33)	4.95 (2.77)	2.66 (1.56)
**Long-term hospital**	2.17 (0.76)	1.0 (1.0)	0.83 (0.76)	8.17 (5.2)	3.0 (1.0)
**Supported accommodation**	2.16 (1.54)	1.1 (1.51)	1.59 (1.38)	5.31 (2.35)	2.32 (1.04)
**Boarding house**	2.53 (1.37)	0.84 (1.04)	1.60 (1.11)	5.77 (2.59)	2.12 (1.01)
**Exercise level** ^**Ф**^					
**Sedentary**	2.35 (1.96)	1.06 (1.47)	1.65 (1.70)	5.30 (2.88)	2.56 (1.70)
**Minimal exercise**	2.66 (1.98)	1.19 (1.63)	1.70 (1.36)	5.41 (2.80)	2.42 (1.23)
**Lightly active**	2.44 (1.53)	1.26 (1.93)	1.60 (1.46)	5.24 (2.62)	2.53 (1.36)
**Moderately active**	2.97 (1.72)	1.74 (1.43)	1.75 (1.53)	5.24 (2.45)	2.97 (2.21)
**Very/extra active**	3.18 (1.83)	1.01 (1.16)	1.61 (1.79)	3.38 (2.21)	3.08 (2.42)
**Smoke status**					
**Never**	2.70 (1.72)	1.41 (1.47)	1.59 (1.29)	5.18 (2.54)	2.47 (1.34)
**Current**	2.31 (1.87)	1.06 (1.83)	1.72 (1.73)	5.39 (2.87)	2.68 (1.79)
**Antipsychotic use**					
**Clozapine + /- other**	2.35 (1.73)	1.22 (1.42)	1.76 (1.56)	5.31 (2.45)	2.43 (1.30)
**Olanzapine + /- other**	2.70 (1.83)	1.26 (1.46)	1.86 (1.82)	5.22 (2.56)	2.46 (1.35)
**Other only**	2.47 (1.82)	1.26 (1.93)	1.55 (1.40)	5.32 (2.89)	2.72 (1.78)
**None**	3.07 (1.91)	1.16 (1.13)	1.52 (1.15)	4.83 (2.82)	2.47 (1.86)
**Diabetes**					
**Yes**	2.40 (1.70)	1.27 (1.53)	1.74 (1.50)	5.50 (2.69)	2.61 (1.86)
**No**	2.55 (1.84)	1.24 (1.72)	1.64 (1.53)	5.19 (2.71)	2.56 (1.48)
**Dyslipidaemia**					
**Yes**	2.50 (1.82)	1.23 (1.69)	1.68 (1.52)	5.28 (2.69)	2.60 (1.59)
**No**	2.62 (1.57)	1.43 (1.51)	1.56 (1.56)	5.07 (2.87)	2.27 (1.59)
**Hypertension**					
**Yes**	2.42 (1.56)	1.29 (1.94)	1.83 (1.61)	5.34 (2.82)	2.57 (1.58)
**No**	2.59 (1.98)	1.20 (1.42)	1.53 (1.44)	5.21 (2.60)	2.58 (1.60)
**Socio-economic status** ^**Ф**^					
**Upper**	2.75 (1.67)	1.38 (1.49)	1.62 (1.21)	5.28 (2.60)	2.74 (1.49)
**Middle**	2.40 (1.28)	1.47 (1.56)	1.67 (1.55)	4.89 (1.73)	2.66 (1.14)
**Lower**	2.40 (1.80)	1.18 (1.74)	1.68 (1.63)	5.28 (2.78)	2.58 (1.64)

Ф Number not equal to 1084.

Univariable regression analysis (Table 2) revealed that male gender, higher BMI, being on a pension, living arrangements, sedentary lifestyle, antipsychotic medication use, smoking, diabetes, and low socio-economic status were statistically significantly associated with intake of vegetables.

**Table 2:** Factors associated with consumption of fruit and vegetable intake (n = 1484).

	Vegetables				Fruits			
	Univariable % change (95% CI)	P value	Multivariable % change (95% CI)	P value	Univariable % change (95% CI)	P value	Multivariable % change (95% CI)	P value
**Age group (years)**								
**≤ 30**	1.8 (-6.6 to 11.1)	ns	-10.9 (-20.1 to -0.8)	0.03	11.7 (1.5 to 22.8)*	0.02	3.7 (-7.0 to 15.7)	ns
**31–50**	0.1 (-5.9 to 6.5)	ns	-4.1 (-10.3 to 2.6)	ns	2.5 (-4.3 to 9.8)	ns	-1.0 (-8.0 to 6.3)	ns
**>50**	Reference				Reference			
**Gender**								
**Male**	-9.7 (-14.8 to -4.3)*	<0.0001	-11.2 (-16.6 to -5.5)*	<0.001	-10.4 (-16.1 to -4.3)*	<0.01	-6.6 (-12.8 to 0.04)*	0.05
								
**Female**	Reference				Reference		Reference	
**BMI**								
**Normal**	Reference				Reference			
**Overweight**	-8.1 (-15.1 to -0.4)*	0.02			8.4 (-0.7 to 18.2)	0.07		
**Obese**	-8.0 (-14.6 to -0.9)	0.04			3.7 (-4.5 to 12.6)	ns		
**Waist-to-height ratio** ^**Ф**^								
**Normal**	Reference				Reference			
**Abnormal**	-2.6 (-10.7 to 6.2)	ns			-7.9 (-16.2 to 1.2)	ns		
**Psychiatric diagnosis**								
**Schizophrenia**	Reference				Reference			
**Schizoaffective**	4.8 (-5.0 to 15.8)	ns			1.3 (-9.3 to 13.0)	ns		
**Bipolar**	0.4 (-8.2 to 9.7)	ns			-1.9 (-11.1 to 8.4)	ns		
**Depression**	13.7 (-3.3 to 33.7)	ns			5.0 (-12.3 to 25.6)	ns		
**Other psychosis**	-0.6 (-10.8 to 10.8)	ns			0.2 (-10.9 to 12.9)	ns		
**All others**	-10.3 (-25.9 to 8.6)	ns			-22.7 (-37.3 to -4.6)	0.01		
**Vocational level**								
**Employed**	Reference		Reference					
**Pension**	-12.3 (-19.0 to -5.0)*	<0.01	-11.0 (-183 to -3.2)*	0.01	-10.0 (-17.5 to -1.7)*	0.01		
**Studying**	-4.4 (-18.0 to 11.5)	ns	-5.2 (-19.0 to 10.8)	ns	6.6 (-9.7 to 25.9)	ns		
**Unemployed**	-4.1 (-13.3 to 6.2)	ns	-5.8 (-15.2 to 4.6)	ns	-1.0 (-11.4 to 10.6)	ns		
**Living arrangements**								
**Alone**	Reference		Reference		Reference			
**Spouse/de facto**	17.3 (5.4 to 30.6)*	<0.01	10.2 (-1.7 to 23.4)*	0.09	19.7 (6.5 to 34.6)*	<0.01	11.8 (-1.1 to 26.4)*	0.07
**Parents or siblings**	11.2 (3.5 to 19.4)	<0.01	9.2 (0.6 to 18.5)	0.03	9.1 (0.8 to 18.1)	0.03	4.4 (-4.4 to 14.1)	ns
**Other family member**	-1.6 (-15.1 to 14.1)	ns	1.9 (-12.7 to 19.0)	ns	-13.1 (-26.2 to 2.2)	ns	-14.3 (-27.6 to 1.4)	0.07
**Group of others**	7.6 (-7.9 to 25.7)	ns	6.9 (-9.2 to 26.0)	ns	-10.7 (-24.9 to 6.01)	0.08	-10.5 (-25.2 to 7.2)	ns
**One other (not family)**	-10.7 (-23.0 to 3.6)	ns	-19.0 (-30.5 to -5.7)	<0.01	15.4 (-1.8 to 35.6)	ns	12.1 (-5.0 to 32.2)	ns
**Supported accommodation**	0.42 (-11.9 to 14.5)	ns	1.2 (-11.7 to 16.0)	ns	-6.7 (-19.0 to 7.5)	ns	-6.3 (-19.0 to 8.4)	ns
**Boarding house**	9.7 (-0.07 to 20.5)	0.05	13.3 (2.8 to 24.8)	0.01	-13.0 (-21.6 to -3.5)	<0.01	-6.2 (-15.6 to 4.3)	ns
**Exercise level** ^**Ф**^								
**Sedentary**	Reference		Reference		Reference		Reference	
**Minimal exercise**	8.2 (0.4 to 16.7)*	0.03	8.3 (0.3 to 16.9)*	0.04	9.3 (1.2 to 18.1)*	0.02	10.1 (1.8 to 19.1)*	0.01
**Lightly active**	9.0 (1.8 to 16.8)	0.01	11.9 (4.4 to 20.1)	<0.001	12.5 (4.7 to 20.9)	<0.01	11.9 (4.0 to 20.4)	<0.01
**Moderately active**	22.4 (11.6 to 34.2)	<0.001	22.2 (11.0 to 34.6)	<0.001	35.0 (22.5 to 48.9)	<0.001	30.2 (17.8 to 43.8)*	<0.001
**Very/extra active**	33.4 (4.5 to 70.3)	0.02	28.3 (-0.6 to 65.5)	0.05	0.7 (-22.7 to 31.3)	ns	-4.5 (-27.6 to 26.0)	ns
**Smoke status**								
**Never**	Reference		Reference		Reference		Reference	
**Current**	-11.9 (-16.7 to -6.9)*	<0.001	-5.6 (-11.1 to 0.3)	0.06	-19.2 (-24.0 to -14.1)*	<0.001	-14.5 (-19.9 to -8.7)*	<0.001
**Antipsychotic use**								
**Clozapine + /- other**	Reference		–		Reference		–	
**Olanzapine + /- other**	7.9 (-0.7 to 17.3)*	0.07			0.7 (-8.2 to 10.5)	ns		
**Other only**	3.7 (-2.9 to 10.7)	ns			-0.004 (-7.1 to 7.6)	ns		
**None**	14.1 (0.6 to 29.3)	0.04			-4.4 (-16.5 to 9.6)	ns		
**Diabetes**								
**Yes**	-6.9 (-12.9 to -0.4)*	0.03	–		-2.2 (-9.1 to 5.3)	ns	–	
**No**	Reference				Reference			
**Dyslipidaemia**								
**Yes**	-4.7 (-14.6 to 6.4)	ns	–		-7.5 (-17.8 to 4.0)	ns	–	
**No**	Reference				Reference			
**Hypertension**								
**Yes**	0.5 (-4.7 to 6.1)	ns	–		0.3 (-5.3 to 6.2)	ns	–	
**No**	Reference				Reference			
**Socio-economic status** ^**Ф**^								
**Upper**	Reference		–		Reference		–	
**Middle**	-11.5 (-17.3 to -5.3)*	ns			3.0 (-15.5 to 25.7)	ns		
**Lower**	-11.1 (-25.4 to 5.8)	<0.001			-10.2 (-16.7 to -3.13)*	<0.01		

*Global p value (Type III test) Statistically significant at p < 0.05.

Older people, male gender, living outside the family, particularly in a boarding house, sedentary lifestyle, being on a pension, low socio-economic status, and smoking showed significant univariable association with low fruit intake (Table 2).

On multivariable regression, males showed 11.2% lower intake of vegetables compared to females. Sedentary behaviour was statistically significantly associated with less consumption of vegetables. Consumers who were on a pension consumed the least serves of vegetables, 11% less compared to consumers who were employed. Those who smoked consumed 5.6% less vegetables compared to non-smokers, however, this difference did not remain significant after adjusting for covariates. Those living with parents or siblings (9.2%) or at a boarding house (13.0%) consumed higher serves of vegetables compared to those living alone.

Multivariable analysis for intake of fruit, showed males consumed 6.6% less fruits compared to females. Those with sedentary behaviour consumed the least serves of fruit and smokers consumed 14.5% less fruit compared to non-smokers. The type III test indicated a significant main effect for living arrangements, however comparisons among the individual levels showed borderline significance.

**Fig 1:**
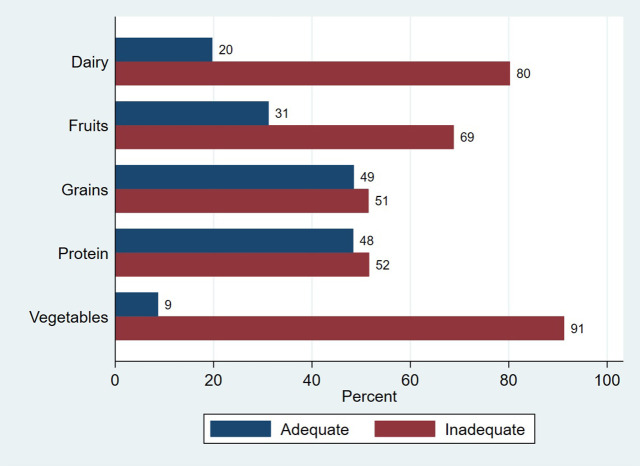
Proportion meeting ADG recommended daily consumption.

In a typical day 7% of patients reported nil consumption of vegetables, 34% reported no fruit intake, 2% no grains intake, 3% no protein intake, and 15% no dairy intake. Most of these patients consumed less than the daily recommended serves for all core food groups. Only 9.0% met the ADG recommendations of eating more than 5–6 serves of vegetables per day, 31.0% ate ≥ 2 serves of fruits, 20.0% ate ≥ 2.5-4 serves of dairy, 48.0% ate ≥ 2–3 serves of protein and 49.0% ate ≥ 4–6 serves of grains (Fig 1).

**Fig 2:**
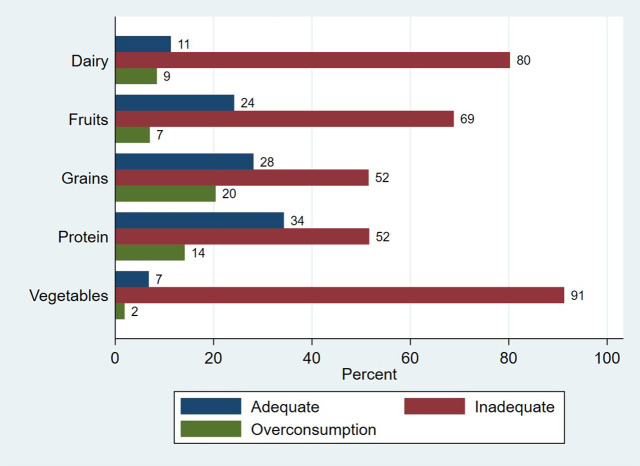
Proportion meeting ADG recommended daily consumption including high intakes.

As shown in Fig 2, majority of patients were consuming inadequate serves according to the ADG recommendations whilst few reported high intakes: vegetables (2.0.%), fruits (7.0%), dairy (9.0%), protein (14.0%) and grains (20.0%). Those in the higher and middle socioeconomic status consumed more fruits compared to the low socioeconomic status (p = 0.02).

Like the Australian general population, people with EPI consumed less than the recommended targets for vegetables and fruits. 51.3% of people in the Australian General Population [22] met the ADG recommendations for fruits vs 31% in people with EPI. For vegetables 7.5% vs 8%. On average people with EPI consumed 2.5 serves of vegetables and 1.2 serves of fruits which is almost half the daily recommended serves by ADG.

According to the dietitian’s report eliciting the reliability of the diet history (essentially a CGI), in half of the occasions (48%), they thought that the consumers were reporting reliable information, in 17% unreliable, and 35% were thought to be providing uncertain information.

**Fig 3:**
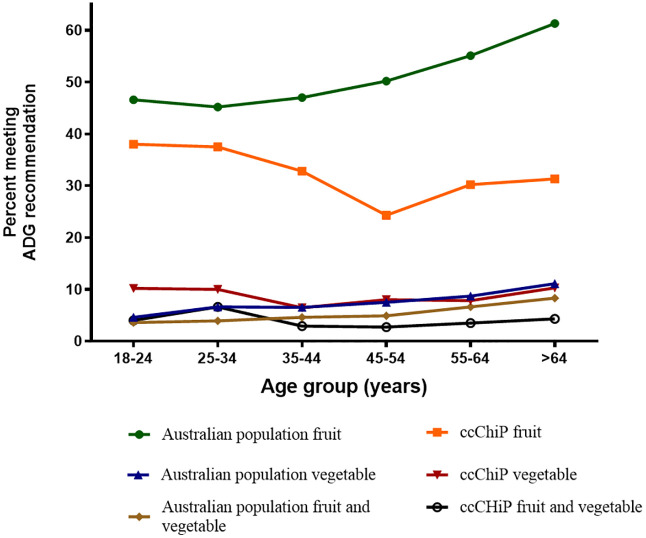
Proportion meeting ADG fruit and vegetable intake recommendations.

Fig 3 shows the trend in vegetable and fruit intake by age groups in people with EPI compared to Australian general population. People with EPI consumed less fruits than the general population across all age groups and the gap between the two populations widens as they get older. However, people with EPI consumed similar amounts of vegetables as the general population, slightly higher in the younger age groups.

**Fig 4:**
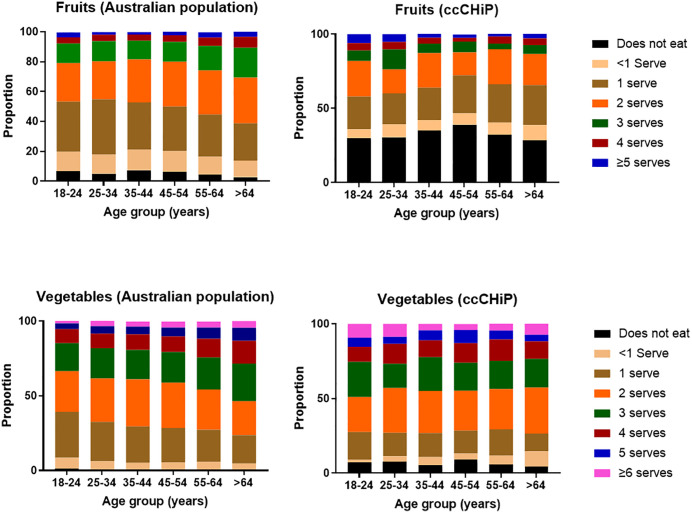
Consumption of fruits and vegetables.

In Fig 4, proportions of serves of fruit and vegetable are compared with the general population data. [30] A greater proportion of people with EPI didn’t consume fruits at all compared to the general population across all age groups (<0.001). Nearly 40% of people with EPI aged 45–54 did not eat any fruits compared to 6% in the matching age group in the general population. Similarly, the proportion of people who didn’t consume any vegetables was greater in the EPI population than those in the general population (9.1% compared with 0.8% in those aged 45–54 years) (p < 0.001).

## Discussion

To our knowledge, this is the first large community-based cohort study in Australia where the intake of core food groups in people with EPI was elicited by a mental health dietitian. Dietary intake patterns of 1084 consumers were assessed. Overall, the results indicate that the mean number of servings for all five of the core food groups were below minimum recommendations. The food group most likely to be consumed in higher amounts (above recommended serves) was grain (cereal) foods (21%). Furthermore, intake of fruit and vegetables was found to be lower in people with EPI compared to the Australian population, which is already below expected minima. On average, Australian adults consumed 3 serves of vegetables and 1.5 serves of fruit [31]. People with EPI consume 2.5 serves of vegetables and 1.2 serves of fruit. Interestingly, the gap for fruit intake compared to the Australian population widens with age. The comparative inadequacies for both fruit and vegetable intake are amplified for those living with EPI by factors such as receiving the disability pension, lower socioeconomic status, living in boarding houses, and smoking [32] which has also been supported by the broader literature [33].

The general poor intake of fruit and vegetables is multifactorial but hypothesised that it may be related to preference for convenience foods (with limited vegetables), regularly skipping meals where fruit is commonly consumed (e.g., breakfast) due to sedation and/or preference of discretionary items as snacks rather than fruit [24].

More than half of our consumers are current smokers and the proportion of people smoking in our study increases with age. Prior evidence has shown that there is an inverse association with smoking and consumption of fruits and vegetables [32,34,35]. This appears consistent with trends observed amongst smokers in our population (Table 2). Furthermore, our findings show that consumption of fruits was inversely associated with smoking and positively linked to socioeconomic status, consistent with other studies [36]. This could be explained by the fact that in our population, more than 90% of people who are smoking ≥ three cigarettes per day are in the ‘lower’ socioeconomic status and their very limited discretionary income may be used to preferentially buy cigarettes. However, further research is needed that includes direct spending data related to food insecurity that allows more accurate understanding of this association. While smoking cessation remains the primary target for the prevention of many chronic diseases, additional attention should also be given to dietary interventions, especially in smokers, as an opportunity to reduce overall cardiometabolic risk.

A significant proportion of participants did not consume any fruit (34%) or vegetables (7%) compared to the general population (Fig 4). This dietary gap in fruit and vegetable consumption may be understood within the context of social determinants of health inequalities, including disparities in income, education level, access to nutritional assessments, health literacy that shape healthy eating. In a British questionnaire-based study that included people with EPI, 8.5% ate no portions of fruit and vegetables per day [26]. Although dietary guidelines vary between countries, most align with WHO recommendations of 400g of fruit and vegetables combined. Identifying this significant gap in fruit intake, amongst people living with EPI, highlights the need for targeted and incremental dietary changes. Meta-analyses [37,38] suggest that each additional daily serve of fruit provides increased protection, reducing coronary heart disease risk by at least 7%. Dietary interventions targeting food groups allows for more positive and practical messaging compared to other more abstract dietary advice, such as to reduce calories, added sugar and sodium intake, which can be hard to independently translate into specific actions and subsequent behavioural change. This is also particularly true when considering the orexigenic effects of psychotropic medications, with increased consumption of fruit and vegetables at meals or as snacks, a key strategy to better manage hunger.

### Strengths

The strengths of the study include its prospective nature and access to people with EPI from a community setting attending a standardised clinical service. This service effectively reaches consumers from community mental health services across a large geographic area accessing both public and private services [39]. Embedding dietitians, within mental-health specific services, provides the unique opportunity to assess, identify and improve modifiable risk factor – such as poor diet. Much of the literature to date has focused on anthropometry and weight loss as a measure of success of dietary interventions, despite a meta-analysis indicating that at best weight loss is minimal to moderate [40]. Future research should be looking to measure diet quality and use of validated tools, specifically within the mental health population.

### Limitations

Comprehensive dietary assessment is clearly limited in clinical practice, with challenges commonly presented in assessing dietary patterns in people with EPI [24]. Dietary intake assessment methods were based on recall and self-report and thus inherently biased and prone to under-reporting. Recall inaccuracies, social desirability bias, and cognitive impairment may have further compromised the accuracy of responses, even with the use of visualisation tools (e.g., realistic food models, colour infographics and so on).

We acknowledge that the reliability rating of the self-reported data we present was gathered in the course of usual clinical practice rather than for research purposes. The intention of collecting this information as a clinical aid is to assist clinicians to manage and assess their physical health needs. We recognise the inherent biases of self-reported diet intake data and the additional limitations introduced by the difficulties in cognition faced by the population at hand. There is a known high likelihood of under/over reporting however it is considered a valid and interesting observational dataset specifically because it is a description of what is found in a ‘live’ clinic. Future studies should include a standardised approach to measure intake in addition to self-report.

Further, we acknowledge that the criteria we have used for our serve sizes are consistent with Australian Dietary Guidelines. The methodology for Australian Health Survey is different, in that this population level nutrition surveillance involves structured interviews that uses a 24-hr recall while ccCHiP uses a dietitian’s diet history that is designed for individual clinical care.

Currently, there are no specific dietary assessment tools that have been validated within an EPI population. As such, the results outlined in this study offer clinically-informed estimates for core foods intake. These findings also do not account for the potential impact of dietary advice and support provided, for those that have previously received or were engaged with dietetic support at the time.

## Conclusion

It has been widely acknowledged that the diet of those with a lived experience of mental illness have a poor diet. This study contributes to the current body of knowledge attempting to establish the extent to which this varies from the general population. Not only are people with EPI failing to consume the number of recommended serves (which are considered the minimum number that should be consumed to reduce risk of diet-related chronic disease), there is a higher proportion of people with EPI that are not regularly consuming any fruit and/or vegetables. Typically, lower fruit and vegetable intake is most evident among EPI patients, who are males, in the lowest income strata, living outside the family -particularly in boarding houses, who are active smokers and have a sedentary lifestyle. Interventions aimed at integrating dietitians into mental health services, development of a validated assessment tool, use of biomarkers as more objective measure of fruit and vegetable intake as well as developing dietary interventions specifically targeting those with high tobacco use and/or living in boarding houses have the potential to improve both physical and mental health outcomes.
